# Diversity and Bias through Receptor–Receptor Interactions in GPCR Heteroreceptor Complexes. Focus on Examples from Dopamine D2 Receptor Heteromerization

**DOI:** 10.3389/fendo.2014.00071

**Published:** 2014-05-13

**Authors:** Kjell Fuxe, Alexander Tarakanov, Wilber Romero Fernandez, Luca Ferraro, Sergio Tanganelli, Malgorzata Filip, Luigi F. Agnati, Pere Garriga, Zaida Diaz-Cabiale, Dasiel O. Borroto-Escuela

**Affiliations:** ^1^Department of Neuroscience, Karolinska Institutet, Stockholm, Sweden; ^2^St. Petersburg Institute for Informatics and Automation, Russian Academy of Sciences, Saint Petersburg, Russia; ^3^Pharmacology Section, Department of Clinical and Experimental Medicine, University of Ferrara, Ferrara, Italy; ^4^Laboratory of Drug Addiction Pharmacology, Department of Pharmacology, Institute of Pharmacology, Polish Academy of Sciences, Kraków, Poland; ^5^Istituto di Ricovero e Cura a Carattere Scientifico, Venice Lido, Italy; ^6^Departament d’Enginyeria Química, Universitat Politècnica de Catalunya, Barcelona, Spain; ^7^Department of Physiology, School of Medicine, University of Málaga, Málaga, Spain

**Keywords:** heterodimerization, G protein-coupled receptor, receptor heterodimers, biased signaling, biased recognition, receptor diversity, receptor–receptor interactions, allosteric modulation

## Abstract

Allosteric receptor–receptor interactions in GPCR heteromers appeared to introduce an intermolecular allosteric mechanism contributing to the diversity and bias in the protomers. Examples of dopamine D2R heteromerization are given to show how such allosteric mechanisms significantly change the receptor protomer repertoire leading to diversity and biased recognition and signaling. In 1980s and 1990s, it was shown that neurotensin (NT) through selective antagonistic NTR–D2 like receptor interactions increased the diversity of DA signaling by reducing D2R-mediated dopamine signaling over D1R-mediated dopamine signaling. Furthermore, D2R protomer appeared to bias the specificity of the NTR orthosteric binding site toward neuromedin N vs. NT in the heteroreceptor complex. Complex CCK2R–D1R–D2R interactions in possible heteroreceptor complexes were also demonstrated further increasing receptor diversity. In D2R–5-HT2AR heteroreceptor complexes, the hallucinogenic 5-HT2AR agonists LSD and DOI were recently found to exert a biased agonist action on the orthosteric site of the 5-HT2AR protomer leading to the development of an active conformational state different from the one produced by 5-HT. Furthermore, as recently demonstrated allosteric A2A–D2R receptor–receptor interaction brought about not only a reduced affinity of the D2R agonist binding site but also a biased modulation of the D2R protomer signaling in A2A–D2R heteroreceptor complexes. A conformational state of the D2R was induced, which moved away from Gi/o signaling and instead favored β-arrestin2-mediated signaling. These examples on allosteric receptor–receptor interactions obtained over several decades serve to illustrate the significant increase in diversity and biased recognition and signaling that develop through such mechanisms.

## Introduction

Receptor diversity through the existence of receptor subtypes for the same transmitter appears to be a general phenomenon among GPCR and ion channel receptors. Receptor diversity in the case of dopamine (DA) transmission is brought about by five DA receptor subtypes, from D1R to D5R and by two isoforms of the D2R: the long form (D2L) and the short form (D2S), two isoforms of the D3R, and several (more than 10) isoforms of D4R; generated by alternative splicing of the same gene (1). The existence of GPCR subtypes, e.g., for DA in a high and low affinity state, probably reflects their role both in volume (extrasynaptic location, low DA concentration) and synaptic (synaptic location, high DA concentration) transmission, respectively. The relevance of receptor–receptor interactions for receptor diversity in the GPCR field was discussed in 1995 based on changes in the affinity of both the high and low affinity state and in the proportion of receptors in the two affinity states induced by the receptor–receptor interactions in the plasma membrane (2). The reciprocal interactions between receptor–receptor interactions and receptor sensitization/desensitization in control of receptor affinity and signaling were also presented (2). The origin of GPCR diversity has since then been expanded by several groups (3). Today the receptor diversity in the GPCR field has been tremendously increased with identification of over 200 GPCR heteromers (4).

## History of Receptor–Receptor Interactions

The early work on the demonstration of neuropeptide–monoamine receptor–receptor interactions in membrane preparations from different regions of the central nervous system (CNS) (5–7) indicated, e.g., the existence of DA receptor subtype-specific interactions with neurotensin (NT) and cholecystokinin (CCK) receptors in putative brain heteroreceptor complexes (8–15). The stronger allosteric NTR–D2R and CCKR–D2R interactions found in sections than in membrane preparations indicated the requirement of intracellular mechanisms and/or a more intact membrane structure for optimal receptor–receptor interactions (16, 17).

### NTR–D2R interactions

Neurotensin was found to reduce the affinity of the high and low affinity D2R agonist binding sites, which correlated with its ability to counteract the DA agonist-induced inhibition of striatal DA and GABA release and to induce neuroleptic actions with relevance for its postulated anti-psychotic actions (2, 10, 18). NT did not modulate the D1 receptor binding characteristics. Thus, by selective antagonistic NTR–D2R interactions NT increases the diversity of DA signaling by changing the pattern of D2R subtype-mediated signaling with D1 like receptor-mediated signaling obtaining a dominant role over D2 like receptor signaling (2). In other words in the striatum through this allosteric receptor–receptor interaction in postulated NTR–D2 like heteroreceptor complexes, a bias had developed in DA transmission toward D1R like vs. D2R like-mediated DA transmission. This was accomplished through a prejunctional allosteric antagonistic NTR–D2R autoreceptor interaction in striatal DA terminals increasing DA release and a postjunctional allosteric antagonistic NTR–D2R interaction mainly located on the cortico-striatal glutamate nerve terminals but also on the striato-pallidal GABA neurons increasing their activity and GABA release.

It was of substantial interest that the C-terminal NT(8–13) fragment also potently and antagonistically modulated rat neostriatal D2Rs (19) and that neuromedin N (NN) also was a potent inhibitory modulator of D2R agonist binding in rat neostriatal membranes (20). In view of the higher potency of NN vs. NT to modulate the affinity of neostriatal D2Rs, in contrast to the higher potency of NT vs. NN to bind to the cloned NTRs (20), the NN-activated neostriatal NT receptors involved in the affinity regulation of the D2Rs, may have developed a bias toward NN vs. NT in terms of affinity and/or potentially efficacy. This may have been accomplished through a reciprocal allosteric D2R–NTR interaction from the D2R protomer biasing the specificity of the NT orthosteric binding site toward NN vs. NT binding in the heteroreceptor complex.

It should be noticed that the antagonistic presynaptic but not the postsynaptic NTR/D2R receptor–receptor interaction was missing in the nucleus accumbens in contrast to dorsal striatum. This led to increases in ventral striatal GABA release as in the dorsal striatum but without increases in DA release (21). Instead, reductions of accumbens DA release were observed likely at least in part related to activation of GABA A receptors on the DA terminals. Thus, regional differences in the microanatomy of the NTR–D2R heteroreceptor complexes can have important regional functional consequences at the local circuit levels of the ventral and dorsal striatum leading to a differential regulation of the striato-pallidal GABA outflow from these two regions by NT. The NT-induced reduction of D2R signaling in the ventral striatum therefore become stronger in the ventral striatum vs. the dorsal striatum, which will favor anti-psychotic actions vs. development of motor side-effects (11, 22).

### CCK2R–D2R receptor interactions

The pharmacological analysis indicated that only the CCK2Rs were involved in the reduction of the affinity of the D2R agonist binding sites in rat striatal membranes, in as much as, e.g., the CCK1 antagonist L364718 was ineffective in counteracting the increase of the *K*_d_ value by 1 nM of CCK-8 (16, 23). Thus, the CCK2R subtype, can selectively interact in an inhibitory way with the D2R agonist recognition, illustrating how receptor subtype development evolving both in the CCK and DA receptor family make possible, e.g., a further diversity modulating the pattern of isoreceptor activity in the DA and CCK receptor systems (2).

Intracerebral microdialysis in the dorsal striatum in combination with studies on DA release, gave *in vivo* correlates to the CCK2R/D2R antagonistic receptor interaction in D2R recognition at the presynaptic level in the striatal DA nerve terminal networks (24). Thus, activation of presumable CCK2Rs may have reduced the D2 autoreceptor affinity contributing to a reduction of the apomorphine-induced inhibition of DA release. Studies on GABA release within the nucleus accumbens supported the existence of an antagonistic CCK2R/D2R interaction also within the postsynaptic cells by the demonstration that CCK-8 (1 μM) increased both GABA and DA release (21). These antagonistic intramembrane interactions involving the postsynaptic D2 like receptors probably take place in the striopallidal GABAergic neurons involving both the dorsal and ventral components of this pathway, the ventral component being of particular interest in relation to schizophrenia in view of its role in controlling the output from the limbic system (11, 22). The D2R is a major target for anti-psychotic drugs.

It was of substantial interest that CCK-8, in contrast, increased the affinity of dopamine for D2Rs in striatal membrane preparations in competition experiments with the D2R like antagonist [^3^H]-raclopride vs. DA (23). The D1 receptor antagonist SCH-23390 blocked this action and restored the ability of CCK-8 to reduce D2R affinity. Thus, it appeared as if D1R activation in the membrane preparations could alter the CCK-8-induced affinity regulation of D2Rs from a reduction to an increase (23). Furthermore, receptor autoradiographic analysis also demonstrated differential modulatory effects of CCK-8 on the affinity of D2Rs, depending on the use of a D2R agonist in saturation experiments or a D2R antagonist in competition experiments using dopamine as a displacer. The explanation for these seemingly contradictory effects of CCK-8 on the D2Rs was proposed to be that CCK-8 reduced the affinity of D2Rs in the absence of D1R activation by dopamine, and increased the affinity of D2Rs in the presence of D1R activation (16). These differential effects could lead to antagonistic and facilitatory allosteric effects on D2R-mediated dopamine transmission, respectively taking place at the plasma membrane level.

Previous work had proposed the existence of mosaics of receptors in plasma membranes (9). The above results opened up the possible existence of a receptor mosaic of D1R, D2R, and CCK2R receptors in striatal and accumbens membrane preparations. The discovery by George, O’Dowd and colleagues (25, 26) of D1R–D2R heteroreceptor complexes in the brain gave strong support to the existence of higher order heteroreceptor complexes composed of CCK2R–D1R–D2R heteroreceptor complexes. In such heteroreceptor complexes coactivation of D1R and D2R protomers may via allosteric receptor–receptor interactions bias the orthosteric binding site of the D2R to respond to the allosteric CCK2R–D2R receptor–receptor interactions with an increase of affinity instead of a reduction of affinity as seen upon CCK2R protomer activation alone. Further studies are needed, for example, using cell lines cotransfected with CCK2R, D2R, and D1R receptor cDNAs to provide direct evidence for the existence of CCK2R–D1R–D2R heterotrimers with these integrative allosteric D1R–D2R–CCK2R receptor–receptor interactions.

## Biased Signaling in GPCRs

In 1995, Kenakin for the first time introduced the pioneering concept of biased agonism or functional selectivity in the GPCR field (27). It was proposed to reflect the agonist selective stabilization of different active states of receptor conformation differentially linked to signaling pathways (27–33). This phenomenon appears to be true for many GPCRs and has an impact on drug discovery (28, 34, 35). GPCRs are regarded as allosteric machines where allosteric modulators exert unique actions and the allosteric effects can be quantified by the Ehlert allosteric model and the Black/Leff operational model (28, 30). The agonist related bias in GPCR systems was mainly introduced through the studies on β-arrestin-mediated signaling and its relevance for drug development (33, 36–40).

In 1995 (2), the concept of allosteric receptor–receptor interactions in putative GPCR heteromers began to be introduced adding an additional allosteric mechanism contributing to the diversity and bias in the GPCR protomers. In recent years, allostery at GPCR homomers and heteromers has been extensively covered and clearly discussed, especially by Smith and Milligan (41). They point out the hallmarks for allosterism, namely that the allosteric modulation is reciprocal, saturable, and shows probe dependence.

The field of D2R heteroreceptor complexes and their relevance for disease has been summarized in recent years (42, 43). The current review will give examples from recent work on D2R heteromers mainly in cellular models indicating how allosteric receptor–receptor interactions in receptor heteromers may increase diversity and bias in the participating receptor protomers of potential relevance for drug development.

## D2R Signaling Dynamics through Allosteric Receptor–Receptor Interactions in D2R–NTR1 Receptor Heteromers

Recent biochemical, histochemical, and co-immunoprecipitation experiments have indicated the existence of antagonistic dopamine D2R and neurotensin 1 (NTR1R) receptor–receptor interactions in the dorsal and ventral striatum using *inter alia* NTR1 receptor antagonists (44, 45). NTR–D3R receptor–receptor interactions may potentially also exist (45). The discovery of the existence of both D2LR–NTR1R and D2SR–NTR1R heteromers was made under basal conditions in living HEK293T cells by means of bioluminescence resonance energy transfer (46). Through confocal laser microscopy, they were also shown to be colocated in the plasma membrane of these cells.

Based on a bioinformatic approach, Tarakanov and Fuxe (47) deduced a set of protriplet aminoacid homologies that contribute to receptor–receptor interactions. This bioinformatic analysis suggests the existence of a basic set of three homology amino acid protriplets (TVM, DLL, and LRA) in the two participating receptor protomers that contribute to the formation of the D2R–NTR1R heteromers by being part of the receptor interface (46).

**Figure 1:**
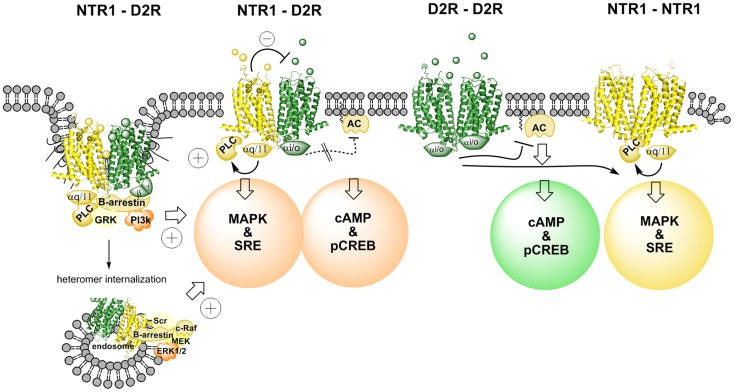
**Illustration of changes in cAMP–pCREB and MAPK–SRE signaling pathways induced by agonist actions at NTR1–D2R heterodimers and at NTR1 receptor homodimers and D2 receptor homodimers**. NT produces via an antagonistic allosteric NTR1–D2 receptor–receptor interaction a reduction in the D2R agonist-induced activation of Gi/o leading to a marked reduction of activity in the cAMP–pCREB pathway. Instead, β-arrestin-mediated internalization and signaling may be favored due to a biased modulation of the D2R protomer via the allosteric receptor–receptor interaction in the heterodimer (see left part). The coagonist treatment also results in a facilitatory interaction in the activation of the MAPK–SRE pathway, which may take place in this signaling pathway at the level of PKC. Thus, the NTR1 agonist can activate the NTR1 protomer, which activates the MAPK–SRE pathway via Gq/11–PLC–PKC. The D2R agonist can target the D2R protomer and produce its activation of the MAPK–SRE pathway via several mechanisms, including β-arrestin–PI3k–PKC or β-arrestin–Scr–PKC (see left part). It should also be noted that this facilitatory interaction can also involve the D2R receptor homodimers and the NTR1 receptor homodimers (see right part). However, the facilitatory interaction may become stronger in the NTR1–D2R heteromer signaling since the NT protomer-induced antagonism of D2R protomer-mediated Gi/o signaling may favor the switch toward β-arrestin recruitment and signaling.

The CREB reporter gene assay indicated that the NTR agonist JMV 449 (10 and 30 nM) markedly reduced the potency of the D2R like agonist quinpirole to inhibit the forskolin-induced increase of the CREB signal (Figure 1). Thus, it seems that the antagonistic allosteric D2R–NTR1 receptor–receptor interaction previously observed on striatal D2R recognition [see review (10)] also exists in the regulation of the D2R/Gi/o coupling inhibiting the adenylyl cyclase (AC) activity, which leads to a reduction of the CREB-mediated signaling (46). These results help explain the antagonistic functional interactions seen with NT and DA ligands in microdialysis and behavioral experiments (48, 49). Thus, the allosteric waves, induced through the NT agonist activation of the orthosteric site of the NTR1 receptor, pass over the receptor interface and transfer the D2R protomer into a conformational state with reduced D2R affinity and Gi/o coupling to the adenylate cyclase.

It is of interest that at 30 nM of the NT agonist used quinpirole could no longer fully counteract the NT agonist action even in the high concentrations. Furthermore, at 50 nM the NT agonist not only counteracted the D2R agonist-induced inhibition but even significantly increased the CREB signal vs. control. These results open up the possibility that the conformational states induced and stabilized in the D2R protomer by the NT agonist via allosteric receptor–receptor interaction also involves a Gs coupling of the D2 agonist quinpirole-activated D2R protomer besides the reduction of the Gi/o coupling (46). Thus, a biased modulation of the signaling pathways of the quinpirole-activated D2R protomer in the D2R–NTR1 heteromer may have developed through the allosteric receptor–receptor interaction (28, 34).

In contrast, the NT agonist was found to markedly increase the quinpirole potency to activate the MAPK pathway as studied with luciferase reporter gene assay measuring the degree of SRE activity as well as with ERK1/2 phosphorylation by means of in cell western assays (Figure 1). The NT receptor agonist-induced enhancement of the D2R agonist-produced increases of MAPK signaling was blocked by the NT receptor antagonist, SR142948. One mechanism for this enhancement of the MAPK signaling of the D2R receptor may be the NT receptor agonist-induced activation of PKC via the Gq–PLC β–PKC pathway (46). Agonist activation of D2Rs is also known to activate PKC involving Gi/o and β-arrestin binding to the D2R and signaling followed by activation of PLC-β (Figure 1). However, this mechanism may be increased in the D2R protomer of the D2R–NTR1 heteromer through the antagonistic allosteric receptor–receptor interaction favoring β-arrestin binding and signaling (see above). Thus, a synergistic activation of PKC by D2R and NTR1R at the level of the signaling cascades may be the mechanism for the ability of agonist coactivation of the D2R and NTR1R to markedly enhance D2R signaling via the MAPK pathway and can involve also the D2 and NTR1 homodimers (Figure 1). In this case, there is likely no allosteric receptor–receptor interaction involved as proposed for the NTR1 protomer-mediated antagonism of the Gi/o signaling from the D2R protomer to AC.

Taken together, the results obtained in this cellular model give strong support to the view that the NTR1 protomer of a D2R–NTR1 heteromer upon activation by a NTR1 agonist via an allosteric receptor–receptor interactions counteracts D2R agonist-induced D2 protomer signaling over Gi/o–AC–PKA–CREB. A biased modulation of the D2R protomer signaling may also develop through this allosteric receptor–receptor interaction. Thus, an increased activity in the CREB pathway over basal activity was observed upon combined treatment with quinpirole and the NTS agonist, which can be explained by a coupling of the D2R protomer to Gs instead of to Gi/o. Instead, the increased activity of the MAPK pathway after coactivation of the D2R and NTR1 receptors likely involves synergistic interactions with PKC at the level of the signaling cascades linked to the heteromer and likely also the D2 and NTR1 homodimers.

The results obtained in the cellular models also have a therapeutic relevance for treatment of schizophrenia since they indicate that the NTR1 protomer in the D2R–NTR1 heteroreceptor complex of the ventral striatum can reduce D2R protomer signaling over the Gi/o–AC–CREB pathway. Thus, development of NTR1 agonists and positive allosteric modulators targeting if possible preferentially the NTR1 protomers represents a relevant strategy. This includes also a potential for reduced side-effects based on the DA hypothesis of schizophrenia and the fact that mainly the D2R protomers in the D2R–NTR1 heteroreceptor complexes are affected (50).

## Actions of Hallucinogenic 5-HT2AR Agonists on the D2R–5-HT2AR Heteroreceptor Complex

Biophysical methods demonstrated dopamine D2R–5-HT2AR heteromers in cellular models after cotransfection of the two receptors (51, 52). This was an interesting finding since atypical anti-psychotic drugs block both D2R and 5-HT2AR receptors (53) and the D2R–5-HT2AR heteroreceptor complexes could therefore represent a novel target for anti-psychotic drugs.

**Figure 2:**
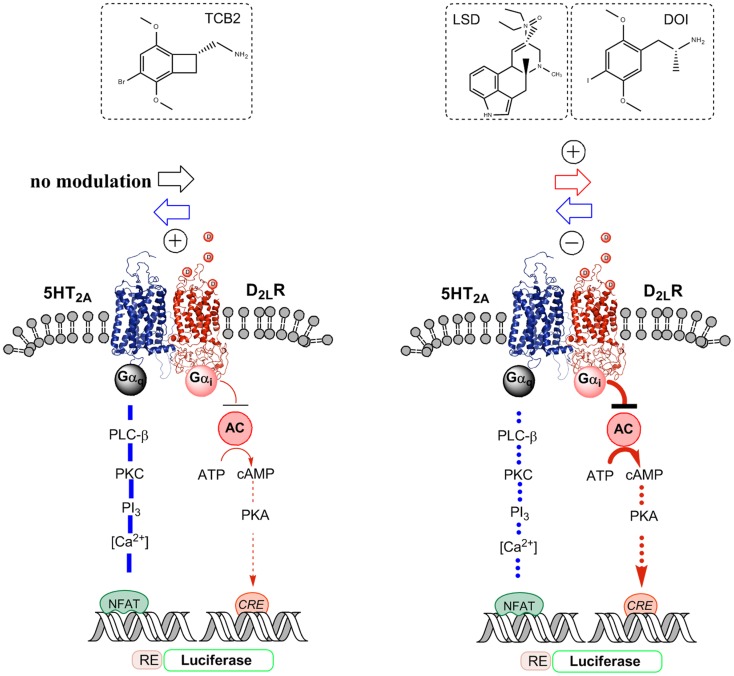
**Illustration of the biased agonist action of the hallucinogenic 5-HT2A agonists LSD and DOI at the 5-HT2A protomer of the 5-HT2A–D2LR heterodimer, which leads to enhanced Gi/o signaling over the D2R protomer producing an enhanced inhibition of the AC–PKA–CREB pathway**. In contrast, the standard 5-HT2A agonist TCB2 did not produce a modulation of the D2R agonist-induced inhibition of the AC–PKA–CREB pathway and the endogenous ligand 5-HT significantly reduced the signaling of this pathway. A biased 5-HT2A agonism with DOI was also observed in the facilitatory D2LR–5-HT2A receptor–receptor interaction of the heteromer, which enhances 5-HT2A signaling over Gq/11–PLC when using 5-HT and TCB2 as 5-HT2A agonists. Thus, when using DOI as a 5-HT2A agonist, a D2R agonist instead diminished 5-HT2A protomer signaling over the Gq/11–PLC pathway.

As studied in HEK-293T27 cells, the 5-HT2AR protomer-mediated phospholipase C (PLC) activation by 5-HT in this heteromer was synergistically enhanced by the concomitant activation of the D2LR protomer by the D2R agonist quinpirole based on an NFAT-luciferase reporter gene assay (Figure 2). A specific and significant elevation of the intracellular calcium levels was also observed when both receptor protomers were simultaneously activated (51). It seems possible that the mechanism involved could be an allosteric D2R–5-HT2AR receptor–receptor interaction increasing the efficacy of the serotonin 5-HT2AR protomer–Gq complex to activate the PLC-initiated cellular signaling pathway. In contrast, using a CRE-luciferase reporter gene assay the D2R protomer-mediated AC inhibition by the D2R agonist quinpirole was reduced by the 5-HT-induced activation of the 5-HT2AR protomer. In this case, the mechanism may be a 5-HT activation of an antagonistic allosteric 5-HT2AR–D2R receptor–receptor interaction, which reduces the efficacy of the quinpirole D2R–Gi/o complex to inhibit the AC-initiated signaling pathway (51). Thus, bidirectional allosteric receptor–receptor interactions may be in operation in this heteromer.

Instead, quinpirole induced activation of the D2R protomer reduces the efficacy of the hallucinogenic 5-HT2AR agonist DOI [(±)-2,5-dimethoxy-4-iodoamphetamine] induced Gq/11 coupling of the 5-HT2AR protomer to the PLC signaling pathway as seen from the diminished production of inositol phosphate by DOI (Figure 2) (54). This is different from the case when the endogenous ligand 5-HT was used to activate the 5-HT2AR (see above). One explanation is that the hallucinogenic 5-HT2AR agonist stabilizes a different active conformation of the 5-HT2AR protomer compared with the endogenous ligand 5-HT, which responds to the quinpirole induced allosteric D2-5–HT2AR receptor–receptor interactions with a reduction in the efficacy of the DOI–5-HT2AR–Gq complex to activate the PLC signaling pathway. Thus, DOI may be a biased agonist at the 5-HT2AR protomer (28, 30).

Based on the above results, we explored if the allosteric counteraction of D2R agonist induced D2R protomer inhibition of the AC signaling pathway through the 5-HT-activated 5-HT2AR protomer was altered when using the known hallucinogenic high affinity 5-HT2AR agonists lysergic acid diethylamide (LSD) and DOI (55). The 5-HT receptor agonist activity of hallucinogens of the indolealkylamine and of the phenylethanolamine type in the CNS was first described in the late 60s and early 70s (56–59). Such an alteration if enhancing D2R protomer function in the D2R–5-HT2AR heteromer may contribute to their psychotic actions. We tested this hypothesis in HEK293-T27 cells and ventral striatum (60) studying 5-HT2AR–D2R interactions using LSD and DOI and a standard 5-HT2AR agonist TCB2 (61) to modulate D2R protomer binding and signaling (Figure 2).

Indications were obtained for the existence of D2R–5-HT2AR heteroreceptor complexes also in discrete regions of the ventral and dorsal striatum with proximity ligation assays (PLA) (60). Using double immunofluorescence procedures with green and red immunofluorescence for D2Rs and 5-HT2ARs, respectively, it was possible to observe a widespread appearance of punctuate yellowish fluorescence in the dorsal striatum and the nucleus accumbens likely representing to a large extent a dendritic colocalization of the two receptors.

The hallucinogenic 5-HT2AR agonists LSD and DOI but not the standard 5-HT2AR agonist TCB2 produced an enhancement of the D2R agonist induced increase of D2R protomer affinity, density, and signaling via Gi/o to inhibit the AC–PKA–CRE pathway (Figure 2). CRE-luciferase reporter gene assays and [^3^H]-raclopride binding experiments were used (60). In contrast, as previously shown the endogenous ligand 5-HT instead caused an antagonistic allosteric 5-HT2AR–D2R receptor–receptor interaction on D2R protomer signaling in these receptor heteromers in HEK293-T27 cells. TCB2 in the range of 10–100 nM, which can fully activate the 5-HT2AR signaling pathways (62) could not modulate the D2R protomer recognition and signaling in [^3^H]-raclopride binding and CRE reporter gene assays, respectively (60). Therefore, the enhancement of D2R signaling over the AC–PKA–CRE pathway produced by LSD and DOI cannot be produced by crosstalk in the signaling cascades of the D2R and 5-HT2AR receptors (Figure 2). The actions of the two hallucinogens were blocked by the 5-HT2AR receptor antagonist ketanserin.

Taken together, the results suggest that the LSD and DOI exert a biased agonist action on the orthosteric site of the 5-HT2AR protomer leading to the development of an active conformational state different from the one produced by 5-HT. This state will then induce a different allosteric 5-HT2AR–D2R receptor–receptor interaction leading not to antagonistic but to facilitatory allosteric interactions, which enhances D2R protomer signaling leading to increased Gi/o-mediated inhibition of the AC–PKA–CRE signaling pathway (Figure 2).

This biased agonist action produced by LSD and DOI at the 5-HT2AR protomer leading to altered receptor–receptor interactions with the D2 protomer is probably also the mechanism for the ability of DOI and LSD to increase the density and agonist affinity of the D2Rs in membranes from D2R and 5-HT2AR cotransfected HEK293-T27 cells and from the ventral striatum, actions blocked by ketanserin. Thus, there may exist cryptic D2R protomers of the D2R–5-HT2AR heteroreceptor complex in the membranes, which cannot bind the D2 like receptor antagonist. However, after the biased agonist action of DOI and LSD an allosteric change develops in the cryptic D2R protomers and their orthosteric binding sites become available to binding by [^3^H]-raclopride. Thus, the biased 5-HT2AR actions of LSD and DOI at the 5-HT2AR protomer lead both to increased D2R density and increased D2R agonist affinity in the high affinity agonist binding site.

In view of these findings, it seems possible that the psychotic actions of the 5-HT2AR agonist hallucinogens (63) can involve enhancement of D2R protomer signaling in the D2R–5-HT2AR heteroreceptor complex in the nucleus accumbens. This may give a novel understanding of the molecular mechanism underlying the therapeutic effects of atypical anti-psychotic drugs. Thus, many atypical anti-psychotics like risperidone and clozapine are *inter alia* characterized by their higher potency to block 5-HT2AR than D2Rs (53, 64–66). It can be speculated that in some forms of schizophrenia this pathological facilitatory 5-HT2AR–D2R interaction has developed in the D2R–5-HT2AR heteroreceptor complex leading to increased D2R protomer recognition and signaling. One advantage of many atypical anti-psychotics can therefore be that they can counteract the D2R protomer signaling at low doses in the D2R–5-HT2AR heteroreceptor complex through their blockade of the 5-HT2AR protomers. This will block the facilitatory receptor–receptor interaction and lead to reduced D2R protomer recognition and signaling. In this way, anti-psychotic effects against positive symptoms of schizophrenia can develop in doses of atypical anti-psychotics that will not fully block several other D2R populations not forming heteroreceptor complexes with 5-HT2AR receptors in the CNS. The blockade of certain of these D2Rs can be involved in producing cognitive and extra-pyramidal side-effects of typical antipsychotics like haloperidol (64).

## Dynamics and Bias through Antagonistic Reciprocal Receptor–Receptor Interactions in A2A–D2 Heteroreceptor Complexes

Adenosine A2A receptor (A2AR)-dopamine D2 receptor heteromerization was demonstrated by means of biochemical and biophysical methods, namely co-immunoprecipitation, bioluminescence resonance energy transfer (BRET), and fluorescence resonance energy transfer analyses, upon transient cotransfection of the two receptors in cell lines (67–71). A2AR–D2R heteroreceptor complexes were later on also found in the striatum using the PLA technique (72, 73).

Antagonistic A2AR–D2R receptor–receptor interactions in striatal membrane preparations were early on demonstrated after incubation with A2AR agonist CGS21680 as seen from demonstrated by the reduction of the affinity of the high affinity D2R agonist-binding site (74). With receptor autoradiography, strong reductions in affinity were observed after A2AR agonist treatment in the nucleus accumbens core and shell of the rat (75). Significant increases in the EC50 values were also observed in the human caudate after incubation of the sections with CGS21680. Furthermore, the antagonistic A2AR–D2R interaction in A2AR–D2R heteromers diminished the Gi/o-mediated signaling of the D2R (71, 76).

In D2R–A2AR cotransfected neuroblastoma cells, coactivation of A2AR and D2R resulted in the coaggregation, cointernalization, and codesensitization of the A2AR and D2R (77). However, it was unknown how the scaffolding protein β-arrestin2, was involved in these events.

Recently it was shown that that the activation of the A2AR–D2R interaction also favors β-arrestin2 recruitment to the D2LR protomer with subsequent increase in cointernalization (68). Thus, this allosteric A2AR–D2R receptor–receptor interaction brings about a biased modulation of the D2 protomer signaling. A conformational state of the D2R protomer is induced, which moves away from Gi/o signaling and instead favors binding of β-arrestin2 and β-arrestin2-mediated signaling (68, 78, 79). This change in functional selectivity can in part explain the reduced time onset of Akt phosphorylation after the A2AR–D2R coactivation followed by a rapid dephosphorylation (68). The decrease in Akt phosphorylation can be caused by the recruitment of PP2A to the D2R–GRK–β-arrestin2–Akt complex (80).

In view of above, the A2AR agonist CGS 21680, which demonstrates atypical anti-psychotic properties (81) may in part exert anti-psychotic properties by increasing β-arrestin2 signaling over the D2R. In agreement, the finding was made in 2011 that β-arrestin-biased dopamine D2R ligands for probing signal transduction pathways is essential for anti-psychotic efficacy (82).

D2R-mediated suppression of NMDA-induced depolarized plateau potential is mediated by the suppression of Cav1.3a L-type calcium channel current induced through the D2R–PLC signaling cascade involving the activation of calcineurin and dephosphorylation of these channels (83). It is of particular interest that also this D2R signaling event can be blocked by A2AR activation likely via the antagonistic allosteric receptor–receptor interaction in the striatal A2AR–D2R heteroreceptor complexes (83). Thus, by means of competitive peptides mimicking the serine-containing epitope it was possible to block the ability of the A2AR to counteract the effects of D2R activation performing perforated-patch-clamp recordings on brain slices.

The glutamate hypothesis of schizophrenia states the existence of a reduced NMDA receptor function in schizophrenia, which may contribute to reduce firing of the ventral striato-pallidal GABA systems contributing to psychotic symptoms. This deficit may at least in part be restored by A2AR agonists. Taken together, the results strongly indicate that biased modulation of the D2R protomer by the A2AR protomer in the A2AR–D2R heteroreceptor complex within the nucleus accumbens can be a promising mechanism for drug development in psychosis.

The existence of an electrostatic interaction between the C-terminal tail of the A2AR and the third intracellular loop (IL3) of the D2R was demonstrated to be importantly involved in the A2AR protomer-mediated allosteric effects on the D2R protomer recognition, signaling, and trafficking (69, 70). Electrostatic bonds of covalent-like strength are formed in this interaction and a detailed mutational analysis was made in the A2AR C-terminal tail (69, 70, 84, 85).

Recently evidence was obtained for the existence of a reciprocal allosteric communication from the D2R to the A2AR protomer of the A2AR–D2R heteromer (86). This allosteric receptor–receptor interaction was found to be mostly mediated by two regions rich in arginine known to give with positive charges, located in IL3 of the D2R. The negative allosteric modulation by the D2R on A2AR agonist binding was shown in a real-time mode. It was possible to determine that D2R activation in part inhibited and also slowed the binding of the fluorescent A2AR agonist to the A2AR (86). The interaction was abolished by mutating the IL3 of the D2R. The Arg residues (217–222 and 267–269) in IL3 of the D2R were demonstrated to play a major role in the antagonistic allosteric D2R–A2AR receptor–receptor interaction. This allosteric receptor–receptor interactions makes possible also an inhibitory modulation by the D2R protomer of the A2AR protomer binding and function.

Taken together, the dynamics through allosteric communication is such that when the A2AR protomer is activated by an A2AR agonist it will not only increase A2AR signaling but also produce a reduction of D2R protomer recognition and a biased modulation of D2R protomer signaling. This will involve a dynamic reduction of D2R protomer-mediated Gi/o signaling and an increase in D2R protomer-mediated β-arrestin2 signaling. Thus, an integrative signal is produced in the receptor heteromer in which the major D2R signal opposing A2AR protomer function is strongly reduced through which a further dominance of A2AR protomer Gs-mediated signaling develops. In the case when the D2R protomer is activated by agonist, an inhibitory allosteric reciprocal D2R–A2AR receptor–receptor interaction develops leading to a reduction of A2AR recognition and signaling. Again an integrative signal develops with a strong D2R protomer signal which through the allosteric communication in the heteromer becomes even more dominant via reduction of the opposing A2AR signal. These events take place rapidly in the plasma membrane. As the A2AR and D2R protomer signals progress into the cytoplasm they will be further integrated in the cytoplasmic signaling cascades, especially the AC–PKA–CRE pathways together with the signals from the A2AR and D2R homodimers (87).

## Biased Recognition in GalR1–GalR2 Heteromers

The three cloned galanin (Gal) receptors show a higher affinity for Gal than for Gal N-terminal fragments like Gal (1–15) (88). An interesting development of this field was the demonstration of specific N-terminal Gal fragment (1–15) binding sites in the rat brain indicating a relevant role of Gal fragments in Gal communication in the CNS, especially in dorsal hippocampus, neocortex, and striatum (89). These areas have a low density of high affinity Gal (1–29) binding sites. Our hypothesis is that these N-terminal Gal fragment preferring sites may be the result of formation of GalR1/GalR2 heteromers. In this heteromer through allosteric modulation, a conformational state is formed in which their Gal recognition sites may be converted into Gal fragment preferring binding sites with reduced affinity for Gal (1–29) (42, 90, 91).

**Figure 3:**
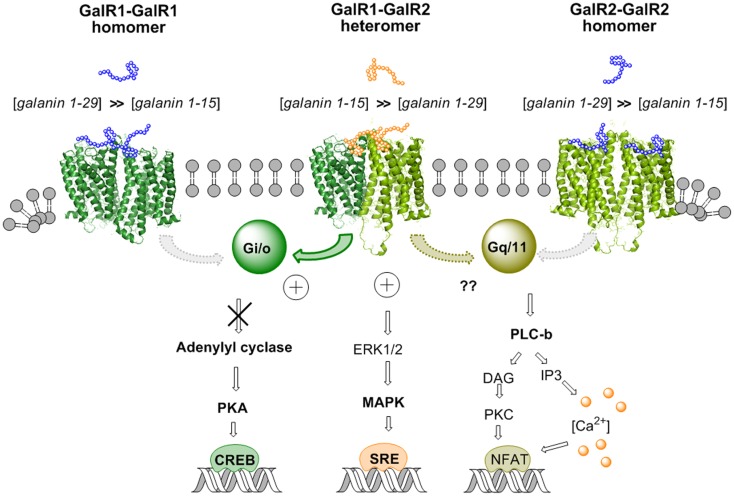
**Illustration of the biased recognition of the GalR1–GalR2 heterodimer**. Thus, this heteromer preferentially recognizes the gal fragment 1–15 vs. galanin 1–29 in contrast to the GalR1 homodimer and the GalR2 homodimer. This is seen from the increased potency of gal 1–15 to reduce activity in the AC–PKA–CREB pathway and increase activity in the MAPK–SRE pathway in cotransfected GalR1 and GalR2 HEK cells vs. GalR1 and GalR2 singly transfected cells. The activity of the GalR2 protomer in the Gq/11–PLC pathway of the GalR–GalR2 heteromer remains to be determined.

Recently it was in fact possible to detect GalR1/GalR2 heteromers in cotransfected Hela cells by *in situ* PLA and in cotransfected HEK293 cells with BRET methodologies (92). A functional validation was also achieved for the existence of GalR1/GalR2 heteromers, which preferentially bind N-terminal Gal (1–15). Using CRE-luciferase reporter gene assays, it was found in GalR1/GalR2 transfected cells that Gal1–15 was significantly more potent than Gal (1–29) in reducing the forskolin-induced increase of luciferase activity through the direct activation of AC (Figure 3). Such evidence was not obtained in singly transfected cells (GalR1 or GalR 2 alone). These results suggest that the agonist activation site of the GalR1–GalR2 heteromer has a preferential affinity for the Gal (1–15) fragment (92). Similar results were obtained in SRE-luciferase reporter gene assays (Figure 3). Gal (1–15) was found to be significantly more potent than Gal1–29 in increasing SRE activity in GalR1 and GalR2 cotransfected cells. This was not the case in singly transfected cells.

Taken together, these results obtained in cellular models open up the possibility that GalR1/GalR2 heteroreceptor complexes exist also in the brain and can be the targets for N-terminal Gal1–15, especially in the hippocampus, neostriatum, and cerebral cortex. Thus, allosteric communication across the receptor interface in the GalR1/GalR2 heteroreceptor complex appears to lead to bias in their orthosteric binding sites toward the Gal1–15 fragment vs. Gal1–29 (Figure 3). This is of special interest for drug development since it was recently reported that Gal1–15 given intraventricularly in the rat produced marked depression-like behavior in the forced swim test and anxiogenic-like effects in the open field (93).

## Concluding Remarks

The allosteric receptor–receptor interactions in the D2 heteroreceptor complexes presented produce upon agonist activation of the partner protomer significant changes in D2R protomer recognition and/or signaling. Thus, this allosteric mechanism through intermolecular interactions changes the D2R protomer repertoire in these complexes diversifying and biasing the D2R function upon agonist activation of the partner protomer. In the case of the NTR–D2R and A2AR–D2R complexes, the NT peptide and A2AR agonist, respectively, both reduced the D2R agonist affinity and reduced the D2R signaling over Gi/o. Not only was the efficacy of the Gi/o signaling reduced in the A2AR–D2R complex but it also switched toward β-arrestin-mediated signaling giving a new bias to the D2R protomer signaling in this complex upon agonist activation of the A2AR protomer. This underlines the view that the D2R protomer can also change and bias its signaling pattern and thus its function by the allosteric receptor–receptor interaction [see also Ref. (25)]. A change in the signaling pattern of the D2R protomer may also have developed in the NTR–D2R complex after high concentrations of NT, in this case toward Gs-mediated D2 protomer signaling. It should be noticed that the NTR and A2AR do not physically interact with the D1 receptors to antagonize their recognition and signaling. Therefore, activation of the NTR and A2A protomers in the D2 heteroreceptor complexes discussed will bias DA signaling in the networks toward D1R-mediated transmission.

The work on the D2R–5-HT2A heteroreceptor complex using 5-HT and the D2like agonist quinpirole illustrated two hallmarks of allostery, reciprocity, and probe dependence. Firstly 5-HT reduced the efficacy of the D2R agonist-activated D2R protomer–Gi/o complex to inhibit the AC–PKA pathway while quinpirole synergistically enhanced the ability of the 5-HT-activated 5-HT2A–Gq complex to activate PLC. The probe dependence was demonstrated by the use of the hallucinogenic 5-HT2A agonists LSD and DOI but not by the standard 5-HT2A agonist TCB2. In this case, the DOI-induced PLC signaling of the 5-HT2A protomer became reduced by quinpirole. Furthermore, DOI and LSD enhanced the D2R agonist quinpirole induced inhibition of the AC–PKA–CRE pathway and increased the density and agonist affinity of the D2 protomer. Thus, LSD and DOI probably induced a biased action on the orthosteric binding site of the 5-HT2A protomer leading to an active conformational state different from the one produced by 5-HT and TCB2. This enhancement of D2R agonist-induced D2R protomer signaling and increase in D2R recognition can contribute to the hallucinogenic and psychotic actions of LSD and DOI in view of the documented role of enhanced D2R recognition and signaling in schizophrenia. In contrast, the agonist activation of the NTR and A2AR protomers reduced the affinity of the D2R protomer and its Gi/o-mediated signaling in the respective heteroreceptor complexes (see above).

CCK2R agonists at the CCK2R protomer of putative CCK2–D2 heteroreceptor complexes also reduced the affinity of the D2R protomer but only in the absence of D1R activation by dopamine. However, this allosteric modulation switched to an increase in the affinity of D2R protomer in the presence of D1R activation. These differential effects illustrated the diversification of the CCK2R allosteric modulation of the D2R protomers into antagonistic or facilitatory allosteric effects dependent on the agonist activation of the D1R. These results can be explained by the existence of higher order heteroreceptor complexes of D1R, D2R, and CCK2R receptors in striatal and accumbens membrane preparations in which the activation of D1R protomers determines the outcome of the allosteric modulation of the D2R protomer. The complex allosteric receptor–receptor interactions in such putative heterotrimeric receptor complexes remain to be determined, which is true also for other higher order heterotrimeric receptor complexes like the A2A–D2–mGluR5 (94) and A2A–D2–CB1 (95). This will determine the impact of the allosteric communication on diversity and bias of the partner protomers and the pattern of signaling that may develop from the receptor protomers of such higher order heteroreceptor complexes.

GalR1/GalR2 heteroreceptor complexes are included to indicate that clearcut-biased recognition can develop in such complexes becoming major targets for N-terminal Gal1–15 in the brain with reduced recognition of Gal. Thus, allosteric communication across the receptor interface in the GalR1/GalR2 heteroreceptor complexes appears to lead to bias in their orthosteric binding sites toward binding the Gal1–15 fragment vs. Gal1–29. They appear to become Gal1–15 receptors and may be named Gal fragment receptors.

## Conflict of Interest Statement

The authors declare that the research was conducted in the absence of any commercial or financial relationships that could be construed as a potential conflict of interest.

## References

[1] Seeman P Historical overview: introduction of the dopamine receptors. In: Neve K, editor. The Dopamine Receptors. New York, NY: Humana Press (2010). p. 1–22

[2] Fuxe K Li XM Tanganelli S Hedlund P O’Connor WT Ferraro L Receptor-receptor interactions and their relevance for receptor diversity. Focus on neuropeptide/dopamine interactions. Ann N Y Acad Sci (1995) 757:365–76 10.1111/j.1749-6632.1995.tb17495.x 7611694

[3] Maudsley S Martin B Luttrell LM The origins of diversity and specificity in g protein-coupled receptor signaling. J Pharmacol Exp Ther (2005) 314:485–94 10.1124/jpet.105.083121 15805429 PMC2656918

[4] Borroto-Escuela DO Brito I Romero-Fernandez W Di Palma M Oflijan J Skieterska K The G protein-coupled receptor heterodimer network (GPCR-HetNet) and its hub components. Int J Mol Sci (2014). (in press). 10.3390/ijms15058570 PMC4057749 24830558

[5] Fuxe K Agnati L Receptor-Receptor Interactions: A New Intramembrane Integrative Mechanism. London: MacMillan Press (1987).

[6] Fuxe K Agnati LF Receptor-receptor interactions in the central nervous system. A new integrative mechanism in synapses. Med Res Rev (1985) 5:441–82 10.1002/med.2610050404 2999530

[7] Fuxe K Agnati LF Benfenati F Celani M Zini I Zoli M Evidence for the existence of receptor – receptor interactions in the central nervous system. Studies on the regulation of monoamine receptors by neuropeptides. J Neural Transm Suppl (1983) 18:165–79 6192208

[8] Fuxe K Li XM Bjelke B Hedlund PB Biagini G Agnati LF Possible mechanisms for the powerful actions of neuropeptides. Ann N Y Acad Sci (1994) 739:42–59 10.1111/j.1749-6632.1994.tb19806.x 7832496

[9] Zoli M Agnati LF Hedlund PB Li XM Ferre S Fuxe K Receptor-receptor interactions as an integrative mechanism in nerve cells. Mol Neurobiol (1993) 7:293–334 10.1007/BF02769180 7514001

[10] Fuxe K Von Euler G Agnati LF Merlo Pich E O’Connor WT Tanganelli S Intramembrane interactions between neurotensin receptors and dopamine D2 receptors as a major mechanism for the neuroleptic-like action of neurotensin. Ann N Y Acad Sci (1992) 668:186–204 10.1111/j.1749-6632.1992.tb27350.x 1361113

[11] Fuxe K Agnati LF Von Euler G Benfenati F Tanganelli S Modulation of dopamine D1 and D2 transmission lines in the central nervous system. In: Osborne N, editor. Current Aspects in the Neurosciences. Basingstoke: MacMillan Press (1990). p. 203–43

[12] Von Euler G Fuxe K Neurotensin reduces the affinity of D-2 dopamine receptors in rat striatal membranes. Acta Physiol Scand (1987) 131:625–6 10.1111/j.1748-1716.1987.tb08285.x 3442244

[13] Agnati LF Fuxe K Benfenati F Battistini N Neurotensin in vitro markedly reduces the affinity in subcortical limbic 3H-N-propylnorapomorphine binding sites. Acta Physiol Scand (1983) 119:459–61 10.1111/j.1748-1716.1983.tb07363.x 6320589

[14] Fuxe K Agnati LF Benfenati F Cimmino M Algeri S Hokfelt T Modulation by cholecystokinins of 3H-spiroperidol binding in rat striatum: evidence for increased affinity and reduction in the number of binding sites. Acta Physiol Scand (1981) 113:567–9 10.1111/j.1748-1716.1981.tb06942.x 6291324

[15] Li XM Hedlund PB Fuxe K Cholecystokinin receptor subtypes regulate dopamine D2 receptors in rat neostriatal membranes. Involvement of D1 receptors. Ann N Y Acad Sci (1994) 713:386–7 10.1111/j.1749-6632.1994.tb44101.x 7910442

[16] Li XM Hedlund PB Fuxe K Cholecystokinin octapeptide in vitro and ex vivo strongly modulates striatal dopamine D2 receptors in rat forebrain sections. Eur J Neurosci (1995) 7:962–71 10.1111/j.1460-9568.1995.tb01084.x 7613631

[17] Li XM Hedlund PB Fuxe K Strong effects of NT/NN peptides on DA D2 receptors in rat neostriatal sections. Neuroreport (1994) 5:1621–4 10.1097/00001756-199408150-00020 7819533

[18] Tanganelli S von Euler G Fuxe K Agnati LF Ungerstedt U Neurotensin counteracts apomorphine-induced inhibition of dopamine release as studied by microdialysis in rat neostriatum. Brain Res (1989) 502:319–24 10.1016/0006-8993(89)90627-6 2819469

[19] Li XM Von Euler G Hedlund PB Finnman UB Fuxe K The C-terminal neurotensin-(8-13) fragment potently modulates rat neostriatal dopamine D2 receptors. Eur J Pharmacol (1993) 234:125–8 10.1016/0014-2999(93)90716-U 8472756

[20] Li XM Finnman UB von Euler G Hedlund PB Fuxe K Neuromedin N is a potent modulator of dopamine D2 receptor agonist binding in rat neostriatal membranes. Neurosci Lett (1993) 155:121–4 10.1016/0304-3940(93)90687-G 8104321

[21] Tanganelli S O’Connor WT Ferraro L Bianchi C Beani L Ungerstedt U Facilitation of GABA release by neurotensin is associated with a reduction of dopamine release in rat nucleus accumbens. Neuroscience (1994) 60:649–57 10.1016/0306-4522(94)90493-6 7936192

[22] Fuxe K Agnati LF Zoli M Pich EM Grimaldi R Bjelke B Neuroplasticity of mesotelencephalic dopamine neurons at the network and receptor level: new aspects of the role of dopamine in schizophrenia and possible pharmacological treatments. In: Tamminga CA Schultz SC, editors. Advances in Neuropsychiatry and Psychopharmacology. New York, NY: Raven Press (1991). p. 51–76

[23] Li XM Hedlund PB Agnati LF Fuxe K Dopamine D1 receptors are involved in the modulation of D2 receptors induced by cholecystokinin receptor subtypes in rat neostriatal membranes. Brain Res (1994) 650:289–98 10.1016/0006-8993(94)91794-9 7953694

[24] Tanganelli S Fuxe K von Euler G Agnati LF Ferraro L Ungerstedt U Involvement of cholecystokinin receptors in the control of striatal dopamine autoreceptors. Naunyn Schmiedebergs Arch Pharmacol (1990) 342:300–4 10.1007/BF00169441 2280797

[25] Rashid AJ So CH Kong MM Furtak T El-Ghundi M Cheng R D1-D2 dopamine receptor heterooligomers with unique pharmacology are coupled to rapid activation of Gq/11 in the striatum. Proc Natl Acad Sci U S A (2007) 104:654–9 10.1073/pnas.0604049104 17194762 PMC1766439

[26] Lee SP So CH Rashid AJ Varghese G Cheng R Lanca AJ Dopamine D1 and D2 receptor co-activation generates a novel phospholipase C-mediated calcium signal. J Biol Chem (2004) 279:35671–8 10.1074/jbc.M401923200 15159403

[27] Kenakin T Agonist-receptor efficacy. II. Agonist trafficking of receptor signals. Trends Pharmacol Sci (1995) 16:232–8 10.1016/S0165-6147(00)89032-X 7667897

[28] Kenakin T Christopoulos A Signalling bias in new drug discovery: detection, quantification and therapeutic impact. Nat Rev Drug Discov (2013) 12:205–16 10.1038/nrd3954 23411724

[29] Luttrell LM Kenakin TP Refining efficacy: allosterism and bias in G protein-coupled receptor signaling. Methods Mol Biol (2011) 756:3–35 10.1007/978-1-61779-160-4_1 21870218

[30] Kenakin T Functional selectivity and biased receptor signaling. J Pharmacol Exp Ther (2011) 336:296–302 10.1124/jpet.110.173948 21030484

[31] Kenakin TP ′7TM receptor allostery: putting numbers to shapeshifting proteins. Trends Pharmacol Sci (2009) 30:460–9 10.1016/j.tips.2009.06.007 19729207

[32] Kenakin T Collateral efficacy in drug discovery: taking advantage of the good (allosteric) nature of 7TM receptors. Trends Pharmacol Sci (2007) 28:407–15 10.1016/j.tips.2007.06.009 17629960

[33] Maudsley S Patel SA Park SS Luttrell LM Martin B Functional signaling biases in G protein-coupled receptors: game theory and receptor dynamics. Mini Rev Med Chem (2012) 12:831–40 10.2174/138955712800959071 22681251 PMC6013268

[34] Kenakin T Miller LJ Seven transmembrane receptors as shapeshifting proteins: the impact of allosteric modulation and functional selectivity on new drug discovery. Pharmacol Rev (2010) 62:265–304 10.1124/pr.108.000992 20392808 PMC2879912

[35] Maudsley S G protein-coupled receptor biased agonism: development towards future selective therapeutics. Mini Rev Med Chem (2012) 12:803 10.2174/138955712800959161 22757722

[36] Luttrell LM Ferguson SS Daaka Y Miller WE Maudsley S Della Rocca GJ Beta-arrestin-dependent formation of beta2 adrenergic receptor-Src protein kinase complexes. Science (1999) 283:655–61 10.1126/science.283.5402.655 9924018

[37] Tohgo A Pierce KL Choy EW Lefkowitz RJ Luttrell LM beta-Arrestin scaffolding of the ERK cascade enhances cytosolic ERK activity but inhibits ERK-mediated transcription following angiotensin AT1a receptor stimulation. J Biol Chem (2002) 277:9429–36 10.1074/jbc.M106457200 11777902

[38] Andresen BT Luttrell LM Brave new world? Arrestin pathway bias in drug design. Endocr Metab Immune Disord Drug Targets (2011) 11:90–1 10.2174/187153011795564142 21599621

[39] Luttrell LM Gesty-Palmer D Beyond desensitization: physiological relevance of arrestin-dependent signaling. Pharmacol Rev (2010) 62:305–30 10.1124/pr.109.002436 20427692 PMC2879915

[40] Gesty-Palmer D Yuan L Martin B Wood WH III Lee MH Janech MG beta-Arrestin-selective G protein-coupled receptor agonists engender unique biological efficacy in vivo. Mol Endocrinol (2013) 27:296–314 10.1210/me.2012-1091 23315939 PMC3683806

[41] Smith NJ Milligan G Allostery at G protein-coupled receptor homo- and heteromers: uncharted pharmacological landscapes. Pharmacol Rev (2010) 62:701–25 10.1124/pr.110.002667 21079041 PMC2993260

[42] Fuxe K Marcellino D Rivera A Diaz-Cabiale Z Filip M Gago B Receptor-receptor interactions within receptor mosaics. Impact on neuropsychopharmacology. Brain Res Rev (2008) 58:415–52 10.1016/j.brainresrev.2007.11.007 18222544

[43] Perreault ML Hasbi A O’Dowd BF George SR Heteromeric dopamine receptor signaling complexes: emerging neurobiology and disease relevance. Neuropsychopharmacology (2014) 39:156–68 10.1038/npp.2013.148 23774533 PMC3857642

[44] Diaz-Cabiale Z Fuxe K Narvaez JA Finetti S Antonelli T Tanganelli S Neurotensin-induced modulation of dopamine D2 receptors and their function in rat striatum: counteraction by a NTR1-like receptor antagonist. Neuroreport (2002) 13:763–6 10.1097/00001756-200205070-00006 11997683

[45] Koschatzky S Tschammer N Gmeiner P Cross-receptor interactions between dopamine D2L and neurotensin NTS1 receptors modulate binding affinities of dopaminergics. ACS Chem Neurosci (2011) 2:308–16 10.1021/cn200020y 22778874 PMC3369761

[46] Borroto-Escuela DO Ravani A Tarakanov AO Brito I Narvaez M Romero-Fernandez W Dopamine D2 receptor signaling dynamics of dopamine D2-neurotensin 1 receptor heteromers. Biochem Biophys Res Commun (2013) 435:140–6 10.1016/j.bbrc.2013.04.058 23624386

[47] Tarakanov AO Fuxe KG Triplet puzzle: homologies of receptor heteromers. J Mol Neurosci (2010) 41:294–303 10.1007/s12031-009-9313-5 19960372

[48] Antonelli T Tomasini MC Fuxe K Agnati LF Tanganelli S Ferraro L Receptor-receptor interactions as studied with microdialysis. Focus on NTR/D2 interactions in the basal ganglia. J Neural Transm (2007) 114:105–13 10.1007/s00702-006-0558-7 16983483

[49] Tanganelli S Antonelli T Tomasini MC Beggiato S Fuxe K Ferraro L Relevance of dopamine D(2)/neurotensin NTS1 and NMDA/neurotensin NTS1 receptor interaction in psychiatric and neurodegenerative disorders. Curr Med Chem (2012) 19:304–16 10.2174/092986712803414268 22335510

[50] Fuxe K Marcellino D Woods AS Giuseppina L Antonelli T Ferraro L Integrated signaling in heterodimers and receptor mosaics of different types of GPCRs of the forebrain: relevance for schizophrenia. J Neural Transm (2009) 116:923–39 10.1007/s00702-008-0174-9 19156349 PMC2953764

[51] Borroto-Escuela DO Romero-Fernandez W Tarakanov AO Marcellino D Ciruela F Agnati LF Dopamine D2 and 5-hydroxytryptamine 5-HT((2)A) receptors assemble into functionally interacting heteromers. Biochem Biophys Res Commun (2010) 401:605–10 10.1016/j.bbrc.2010.09.110 20888323

[52] Lukasiewicz S Polit A Kedracka-Krok S Wedzony K Mackowiak M Dziedzicka-Wasylewska M Hetero-dimerization of serotonin 5-HT(2A) and dopamine D(2) receptors. Biochim Biophys Acta (2010) 1803:1347–58 10.1016/j.bbamcr.2010.08.010 20831885

[53] Meltzer HY Matsubara S Lee JC Classification of typical and atypical antipsychotic drugs on the basis of dopamine D-1, D-2 and serotonin2 pKi values. J Pharmacol Exp Ther (1989) 251:238–46 2571717

[54] Albizu L Holloway T Gonzalez-Maeso J Sealfon SC Functional crosstalk and heteromerization of serotonin 5-HT2A and dopamine D2 receptors. Neuropharmacology (2011) 61:770–7 10.1016/j.neuropharm.2011.05.023 21645528 PMC3556730

[55] Titeler M Lyon RA Glennon RA Radioligand binding evidence implicates the brain 5-HT2 receptor as a site of action for LSD and phenylisopropylamine hallucinogens. Psychopharmacology (1988) 94:213–6 10.1007/BF00176847 3127847

[56] Anden NE Corrodi H Fuxe K Hokfelt T Evidence for a central 5-hydroxytryptamine receptor stimulation by lysergic acid diethylamide. Br J Pharmacol (1968) 34:1–7 10.1111/j.1476-5381.1968.tb07943.x 5302837 PMC1703426

[57] Anden NE Corrodi H Fuxe K Meek JL Hallucinogenic phenylethylamines: interactions with serotonin turnover and receptors. Eur J Pharmacol (1974) 25:176–84 10.1016/0014-2999(74)90047-8 4435021

[58] Anden NE Corrodi H Fuxe K Hallucinogenic drugs of the indolealkylamine type and central monoamine neurons. J Pharmacol Exp Ther (1971) 179:236–49 5133600

[59] Fuxe K Holmstedt B Jonsson G Effects of 5-methoxy-N,N-dimethyltryptamine on central monoamine neurons. Eur J Pharmacol (1972) 19:25–34 10.1016/0014-2999(72)90073-8 4403108

[60] Borroto-Escuela DO Romero-Fernandez W Narvaez M Oflijan J Agnati LF Fuxe K Hallucinogenic 5-HT2AR agonists LSD and DOI enhance dopamine D2R protomer recognition and signaling of D2-5-HT2A heteroreceptor complexes. Biochem Biophys Res Commun (2014) 443:278–84 10.1016/j.bbrc.2013.11.104 24309097

[61] Fox MA French HT LaPorte JL Blackler AR Murphy DL The serotonin 5-HT(2A) receptor agonist TCB-2: a behavioral and neurophysiological analysis. Psychopharmacology (2010) 212:13–23 10.1007/s00213-009-1694-1 19823806

[62] McLean TH Parrish JC Braden MR Marona-Lewicka D Gallardo-Godoy A Nichols DE 1-Aminomethylbenzocycloalkanes: conformationally restricted hallucinogenic phenethylamine analogues as functionally selective 5-HT2A receptor agonists. J Med Chem (2006) 49:5794–803 10.1021/jm060656o 16970404

[63] Colpaert FC Discovering risperidone: the LSD model of psychopathology. Nat Rev Drug Discov (2003) 2:315–20 10.1038/nrd1062 12669030

[64] Meltzer HY Update on typical and atypical antipsychotic drugs. Annu Rev Med (2013) 64:393–406 10.1146/annurev-med-050911-161504 23020880

[65] Meltzer HY Serotonergic mechanisms as targets for existing and novel antipsychotics. Handb Exp Pharmacol (2012) 212:87–124 23129329 10.1007/978-3-642-25761-2_4

[66] Miyamoto S Duncan GE Marx CE Lieberman JA Treatments for schizophrenia: a critical review of pharmacology and mechanisms of action of antipsychotic drugs. Mol Psychiatry (2005) 10:79–104 15289815 10.1038/sj.mp.4001556

[67] Kamiya T Saitoh O Yoshioka K Nakata H Oligomerization of adenosine A2A and dopamine D2 receptors in living cells. Biochem Biophys Res Commun (2003) 306:544–9 10.1016/S0006-291X(03)00991-4 12804599

[68] Borroto-Escuela DO Romero-Fernandez W Tarakanov AO Ciruela F Agnati LF Fuxe K On the existence of a possible A2A-D2-beta-arrestin2 complex: A2A agonist modulation of D2 agonist-induced beta-arrestin2 recruitment. J Mol Biol (2011) 406:687–99 10.1016/j.jmb.2011.01.022 21256133

[69] Borroto-Escuela DO Romero-Fernandez W Tarakanov AO Gomez-Soler M Corrales F Marcellino D Characterization of the A2AR-D2R interface: focus on the role of the C-terminal tail and the transmembrane helices. Biochem Biophys Res Commun (2010) 402:801–7 10.1016/j.bbrc.2010.10.122 21040702

[70] Borroto-Escuela DO Marcellino D Narvaez M Flajolet M Heintz N Agnati L A serine point mutation in the adenosine A2AR C-terminal tail reduces receptor heteromerization and allosteric modulation of the dopamine D2R. Biochem Biophys Res Commun (2010) 394:222–7 10.1016/j.bbrc.2010.02.168 20197060

[71] Fuxe K Agnati LF Jacobsen K Hillion J Canals M Torvinen M Receptor heteromerization in adenosine A2A receptor signaling: relevance for striatal function and Parkinson’s disease. Neurology (2003) 61:S19–23 10.1212/01.WNL.0000095206.44418.5C 14663004

[72] Trifilieff P Rives ML Urizar E Piskorowski RA Vishwasrao HD Castrillon J Detection of antigen interactions ex vivo by proximity ligation assay: endogenous dopamine D2-adenosine A2A receptor complexes in the striatum. Biotechniques (2011) 51:111–8 10.2144/000113719 21806555 PMC3642203

[73] Borroto-Escuela DO Romero-Fernandez W Garriga P Ciruela F Narvaez M Tarakanov AO G protein-coupled receptor heterodimerization in the brain. Methods Enzymol (2013) 521:281–94 10.1016/B978-0-12-391862-8.00015-6 23351745

[74] Fuxe K Ferre S Zoli M Agnati LF Integrated events in central dopamine transmission as analyzed at multiple levels. Evidence for intramembrane adenosine A2A/dopamine D2 and adenosine A1/dopamine D1 receptor interactions in the basal ganglia. Brain Res Brain Res Rev (1998) 26:258–73 10.1016/S0165-0173(97)00049-0 9651540

[75] Diaz-Cabiale Z Hurd Y Guidolin D Finnman UB Zoli M Agnati LF Adenosine A2A agonist CGS 21680 decreases the affinity of dopamine D2 receptors for dopamine in human striatum. Neuroreport (2001) 12:1831–4 10.1097/00001756-200107030-00014 11435907

[76] Fuxe K Marcellino D Genedani S Agnati L Adenosine A(2A) receptors, dopamine D(2) receptors and their interactions in Parkinson’s disease. Mov Disord (2007) 22:1990–2017 10.1002/mds.21440 17618524

[77] Hillion J Canals M Torvinen M Casado V Scott R Terasmaa A Coaggregation, cointernalization, and codesensitization of adenosine A2A receptors and dopamine D2 receptors. J Biol Chem (2002) 277:18091–7 10.1074/jbc.M107731200 11872740

[78] Fuxe K Borroto-Escuela DO Romero-Fernandez W Palkovits M Tarakanov AO Ciruela F Moonlighting proteins and protein-protein interactions as neurotherapeutic targets in the g protein-coupled receptor field. Neuropsychopharmacology (2014) 39:131–55 10.1038/npp.2013.242 24105074 PMC3857668

[79] Borroto-Escuela DO Tarakanov AO Guidolin D Ciruela F Agnati LF Fuxe K Moonlighting characteristics of G protein-coupled receptors: focus on receptor heteromers and relevance for neurodegeneration. IUBMB Life (2011) 63:463–72 10.1002/iub.473 21698749

[80] Beaulieu JM Gainetdinov RR Caron MG The Akt-GSK-3 signaling cascade in the actions of dopamine. Trends Pharmacol Sci (2007) 28:166–72 10.1016/j.tips.2007.02.006 17349698

[81] Rimondini R Ferre S Ogren SO Fuxe K Adenosine A2A agonists: a potential new type of atypical antipsychotic. Neuropsychopharmacology (1997) 17:82–91 10.1016/S0893-133X(97)00033-X 9252983

[82] Allen JA Yost JM Setola V Chen X Sassano MF Chen M Discovery of beta-arrestin-biased dopamine D2 ligands for probing signal transduction pathways essential for antipsychotic efficacy. Proc Natl Acad Sci U S A (2011) 108:18488–93 10.1073/pnas.1104807108 22025698 PMC3215024

[83] Azdad K Gall D Woods AS Ledent C Ferre S Schiffmann SN Dopamine D2 and adenosine A2A receptors regulate NMDA-mediated excitation in accumbens neurons through A2A-D2 receptor heteromerization. Neuropsychopharmacology (2009) 34:972–86 10.1038/npp.2008.144 18800071 PMC5527972

[84] Woods AS Ciruela F Fuxe K Agnati LF Lluis C Franco R Role of electrostatic interaction in receptor-receptor heteromerization. J Mol Neurosci (2005) 26:125–32 10.1385/JMN:26:2-3:125 16012186

[85] Ciruela F Burgueno J Casado V Canals M Marcellino D Goldberg SR Combining mass spectrometry and pull-down techniques for the study of receptor heteromerization. Direct epitope-epitope electrostatic interactions between adenosine A2A and dopamine D2 receptors. Anal Chem (2004) 76:5354–63 10.1021/ac049295f 15362892

[86] Fernandez-Duenas V Gomez-Soler M Jacobson KA Kumar ST Fuxe K Borroto-Escuela DO Molecular determinants of A2AR-D2R allosterism: role of the intracellular loop 3 of the D2R. J Neurochem (2012) 123:373–84 10.1111/j.1471-4159.2012.07956.x 22924752 PMC3480334

[87] Herrick-Davis K Grinde E Cowan A Mazurkiewicz JE Fluorescence correlation spectroscopy analysis of serotonin, adrenergic, muscarinic, and dopamine receptor dimerization: the oligomer number puzzle. Mol Pharmacol (2013) 84:630–42 10.1124/mol.113.087072 23907214 PMC3781380

[88] Branchek T Smith KE Walker MW Molecular biology and pharmacology of galanin receptors. Ann N Y Acad Sci (1998) 863:94–107 10.1111/j.1749-6632.1998.tb10687.x 9928163

[89] Hedlund PB Yanaihara N Fuxe K Evidence for specific N-terminal galanin fragment binding sites in the rat brain. Eur J Pharmacol (1992) 224:203–5 10.1016/0014-2999(92)90806-F 1281778

[90] Fuxe K Borroto-Escuela DO Romero-Fernandez W Tarakanov AO Calvo F Garriga P On the existence and function of galanin receptor heteromers in the central nervous system. Front Endocrinol (2012) 3:127 10.3389/fendo.2012.00127 23112793 PMC3481144

[91] Borroto-Escuela DO Narvaez M Marcellino D Parrado C Narvaez JA Tarakanov AO Galanin receptor-1 modulates 5-hydroxytryptamine-1A signaling via heterodimerization. Biochem Biophys Res Commun (2010) 393:767–72 10.1016/j.bbrc.2010.02.078 20171159

[92] Borroto-Escuela DO Calvo F Narvaez M Romero-Fernandez W Tena M Agnati LF The GalR1-GalR2 heteroreceptor complex preferentially recognizing galanin fragment 1-15. Biochem Biophys Res Commun (2014). 10.1016/j.bbrc.2014.08.061 25152404

[93] Millon C Flores A Narvaez M Parrado C Pulgcerver A Covenas R Galanin N-Terminal Fragment(1-15) Produces Depressant and Anxiogenic Like Actions in the Rat Forced Swimming Test and Open Field Test. Barcelona: FENS Congress FENS (2012). 936 p.

[94] Cabello N Gandia J Bertarelli DC Watanabe M Lluis C Franco R Metabotropic glutamate type 5, dopamine D2 and adenosine A2a receptors form higher-order oligomers in living cells. J Neurochem (2009) 109:1497–507 10.1111/j.1471-4159.2009.06078.x 19344374 PMC3925975

[95] Carriba P Navarro G Ciruela F Ferre S Casado V Agnati L Detection of heteromerization of more than two proteins by sequential BRET-FRET. Nat Methods (2008) 5:727–33 10.1038/nmeth.1229 18587404

